# *KCNH2*‐L693P Causes Long QT Syndrome Type 2 Through hERG Channel Dysfunction: Functional Validation of a Variant of Uncertain Significance

**DOI:** 10.1002/mgg3.70155

**Published:** 2025-11-17

**Authors:** Xi‐Fan Zheng, Qiu Chen, Xiang‐Ting Lu, Hai‐Long Dai, Xue‐Feng Guang

**Affiliations:** ^1^ Kunming Medical University Kunming People's Republic of China; ^2^ Department of Cardiology, Yan'an Hospital Affiliated to Kunming Medical University Kunming People's Republic of China

**Keywords:** congenital long QT syndrome, gene mutation, hERG, *KCNH2‐*L693P, pathogenicity

## Abstract

**Background:**

Congenital long QT syndrome (LQTS) is an inherited arrhythmia characterized by QT prolongation and increased risk of ventricular arrhythmias. Type 2 LQTS (LQT2) results from mutations in the *KCNH2* gene encoding the hERG potassium channel. With the widespread use of next‐generation sequencing, many *KCNH2* variants have been identified but remain classified as variants of uncertain significance (VUS), including p.L693P, requiring functional validation.

**Methods:**

A proband with recurrent syncope and prolonged QTc underwent whole‐exome and family‐based Sanger sequencing, which detected a heterozygous *KCNH2*‐L693P mutation. HEK293 cells were transiently transfected with wild‐type (WT) and/or mutant hERG plasmids. Western blotting, immunofluorescence, and whole‐cell patch clamp were performed to assess protein maturation, trafficking, and channel kinetics.

**Results:**

Western blot showed reduced levels of the 155 kDa mature hERG and accumulation of the 135 kDa immature form, consistent with trafficking defects. Immunofluorescence confirmed endoplasmic reticulum (ER) retention. Electrophysiology revealed complete current loss in homozygotes and ~39% residual WT current in heterozygotes, indicating dominant‐negative‐like suppression. The mutation shifted steady‐state inactivation and delayed recovery.

**Conclusions:**

The *KCNH2*‐L693P mutation impairs hERG maturation and function, supporting its reclassification from variants of uncertain significance (VUS) to pathogenic and providing evidence for improved clinical management.

## Graphical abstract

This study functionally confirmed the pathogenicity of the KCNH2 missense variant L693P, previously classified as a variant of uncertain significance, which causes type 2 congenital long QT syndrome by impairing potassium channel maturation and altering inactivation kinetics.

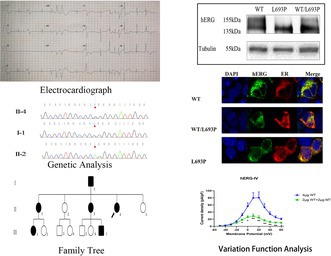

## 1 Introduction

Congenital long QT syndrome (LQTS) encompasses a group of inherited cardiac arrhythmia disorders characterized primarily by a prolonged QT interval on the electrocardiogram (ECG) (Ono et al. 2020). Clinically, it is presented with symptoms such as syncope, palpitations, and seizures, and in severe cases, leads to Torsades de Pointes and sudden cardiac death (Wang et al. 2012). To date, mutations in at least 17 genes have been implicated in the etiology of LQTS (Wilde et al. 2022). Among these, long QT syndrome type 2 (LQT2), resulting from mutations in the *KCNH2* (OMIM#152427) gene, represents one of the most common and clinically significant subtypes (Splawski et al. 2000). Patients with LQT2 demonstrate substantial QT prolongation and an increased susceptibility to life‐threatening arrhythmias triggered by emotional stress or sudden fright (Schwartz et al. 2001).

*KCNH2* gene encodes the human Ether‐à‐go‐go‐Related Gene (hERG) potassium channel, a tetrameric protein essential for cardiac repolarization (MacKinnon 1991). Specifically, hERG is the pore‐forming subunit that forms the rapidly activating delayed rectifier potassium current (IKr), which is critical for repolarizing the cardiomyocytes during the action potential (AP) (Sanguinetti et al. 1996). *KCNH2* mutations dysregulate the function of the hERG potassium channel, leading to a reduction in IKr, disruption of repolarization, prolonged QT interval, and increased risk of ventricular arrhythmias (Al‐Moubarak et al. 2020; Mihic et al. 2011). Mechanistically, *KCNH2* mutations cause impaired hERG channel synthesis, defective intracellular trafficking, aberrant channel gating, or altered ion permeability (Smith et al. 2016). Previous studies have elucidated pathogenic mechanisms associated with several *KCNH2* mutations. However, many *KCNH2* variants, including the *KCNH2*‐L693P variant, are still classified as variants of uncertain significance (VUS) (Anderson et al. 2014; Kapplinger et al. 2009), thereby underscoring the need for further investigation.

This study investigated the pathogenic mechanism of a VUS in a patient presenting with classical clinical features of LQTS.

## 2 Materials and Methods

### 2.1 Ethics Compliance

This study was approved by the Ethics Committee of Yan'an Hospital, affiliated with Kunming Medical University (Approval No. 2022‐190‐01). All the subjects participating in this study signed an informed consent form. This study was conducted according to the Declaration of Helsinki guidelines.

### 2.2 Whole Exome Sequencing, Sanger Validation, and Clinical Evaluation

The patient underwent a standardized physical examination, electrocardiography, echocardiography, electrolyte testing, and medical history evaluation. A peripheral blood sample was collected and sent to the Beijing Nuo He Xin Kang Science and Technology Co. Ltd., for analysis. To screen for gene mutations related to long QT syndrome, whole exome sequencing was performed using target sequence capture high‐throughput sequencing technology on the Illumina NovaSeq series sequencing platform. Sequence variants were annotated according to the GenBank reference sequence of *KCNH2* (NM_000238.4; genome assembly GRCh37/hg19). Sanger sequencing of the identified *KCNH2* variant was performed in all available family members to confirm the mutation and assess its segregation.

### 2.3 Plasmid Construction and Sequencing

The pcDNA3.0‐WT‐hERG plasmid and the pcDNA3.0‐L693P‐hERG plasmid (encoding *KCNH2* wild‐type or *KCNH2* p.L693P, respectively) were commercially purchased from Shanghai GeneChem Company. The plasmids were amplified into the competent *Escherichia coli* , and the plasmid DNA extraction was performed using the PureLink HiPure Plasmid Extraction kit (Invitrogen, K210007, Thermo Fisher Scientific, Waltham, MA, USA). The concentration of plasmid DNA was measured using a DeNovix DS‐11 spectrophotometer (DeNovix Inc., Wilmington, DE, USA). All the plasmids used in the experiments were sequenced and verified by the Shanghai GeneChem Company.

### 2.4 Cell Culture and Transient Transfection

HEK 293 cells were cultured with 293 cell complete medium (Pricella, CM‐0001, Wuhan, Hubei, China) in 35 mm culture dishes and used for subsequent experiments. HEK 293 cells were transiently transfected with different plasmids using Lipofectamine 3000 (Invitrogen, Thermo Fisher Scientific, Waltham, MA, USA) according to the manufacturer's instructions. For western blotting (WB) and whole‐cell patch clamp experiments, a non‐fusion GFP‐expressing plasmid was used for transient transfections, and the following groups were established: (1) WT group (4 μg or 2 μg of WT‐hERG plasmid); (2) homozygous mutant group (4 μg of L693P‐hERG mutant plasmid); and (3) heterozygous mutant group (2 μg of WT‐hERG plus 2 μg of L693P‐hERG mutant plasmid). After transient transfections, the cells were cultured for 48 h before being used for WB and patch clamp experiments. For the immunofluorescence experiments, transient transfections were performed with the WT or mutant *KCNH2* cloned into a plasmid containing a GFP reporter gene and a reporter plasmid containing an endoplasmic reticulum marker protein fused to a red fluorescence protein. Subsequently, we generated the following experimental groups: (1) WT group (4 μg of WT‐hERG plasmid and 4 μg of pDsRed2‐ER plasmid); (2) homozygous mutant group (4 μg of L693P‐hERG mutant plasmid and 4 μg of pDsRed2‐ER plasmid); and (3) heterozygous mutant group (2 μg of WT‐hERG, 2 μg of L693P‐hERG mutant plasmid, and 4 μg of pDsRed2‐ER plasmid). After transient transfection, the cells were further cultured for 48 h before analysis by confocal microscopy.

### 2.5 Western Blot

After 48 h of transient transfection, total cellular protein was extracted using the RIPA lysis buffer (Beyotime, Biotechnology, Suzhou, China) containing 1% PMSF (Beyotime, Biotechnology, Suzhou, China) and 2% phosphatase inhibitors (MedChemExpress, Shanghai, China). The protein concentration was measured using the BCA protein assay kit (Beyotime, Biotechnology, Suzhou, China). Equal amounts of protein lysates were denatured in loading buffer (Beyotime, Biotechnology, Suzhou, China) at 60°C for 8 min (Anderson et al. 2006). Then, the proteins were separated by electrophoresis on a 7.5% SDS‐PAGE gel (Epizyme Biotech, Shanghai, China) and subsequently transferred onto a PVDF membrane (Millipore, Burlington, MA, USA). The membrane was then blocked at room temperature with the BSA blocking solution (TBST +5% BSA) for 1 h. Subsequently, the membrane was incubated overnight at 4°C with the following primary antibodies: *KCNH2* (Alomone Company, APC‐109, Jerusalem, Israel) and Tubulin (Immunoway Company, PT282, Plano, TX, USA). Then, the membrane was incubated with an HRP‐conjugated secondary antibody (Proteintech Group, Wuhan, China) at room temperature for 1 h. The protein bands were developed using ECL (Proteintech Group, Wuhan, China) and visualized.

### 2.6 Confocal Microscopy

After 48 h of transient transfection, the cells from different experimental groups were harvested, loaded on slides, and imaged using a Nikon confocal laser scanning microscope (NIKON C2, Nikon Corporation, Tokyo, Japan). Fluorescence signals were detected with the following excitation and emission settings: nuclear staining with DAPI (Ex 405 nm, Em 417–477 nm), hERG fused with green fluorescent protein (GFP; Ex 488 nm, Em 500–550 nm), and endoplasmic reticulum marker pDsRed2‐ER (Ex 561 nm, Em 570–1000 nm). Sequential scanning mode was applied to avoid channel cross‐talk.

### 2.7 Whole‐Cell Patch‐Clamp Electrophysiological Recording

After 48 h of transfection, hERG currents were recorded in different groups of HEK293 cells using the whole‐cell patch‐clamp technique. The data were acquired using an EPC‐10 amplifier (HEKA Elektronik GmbH, Lambrecht, Germany) and stored using the PatchMaster software (HEKA Elektronik GmbH, Lambrecht, Germany). The sampling rate was set at 20.0 kHz. The signals were filtered at 2.9 kHz. All the experiments were performed at room temperature. The holding potential was maintained at −80 mV. Specific voltage‐clamp protocols (details in the results section) were used to investigate the activation, deactivation, and inactivation properties of the hERG currents.

Recording pipettes were fabricated from borosilicate glass capillaries (BF150‐86‐10, Sutter Instrument, Novato, CA, USA) using a horizontal puller (P‐97, Sutter Instrument, Novato, CA, USA). When filled with the internal solution, pipette tip resistance was 2–5 MΩ. During whole‐cell recordings, the access (series) resistance was monitored and maintained below 15 MΩ. The offset potential generated by potassium aspartate in the pipette solution (~115 mM) was not corrected.

The extracellular solution contained 140 mM NaCl, 3.5 mM KCl, 1 mM MgCl_2_·6H_2_O, 2 mM CaCl_2_·2H_2_O, 10 mM D‐glucose, 10 mM HEPES, and 1.25 mM NaH_2_PO_4_·2H_2_O, and the pH was adjusted to 7.4 with NaOH. The intracellular solution included 20 mM KCl, 115 mM potassium aspartate, 1 mM MgCl_2_·6H_2_O, 5 mM EGTA, 10 mM HEPES, and 2 mM Na_2_‐ATP, and the pH was adjusted to 7.2 with KOH.

### 2.8 Statistical Analysis

The data from the whole‐cell patch‐clamp experiments were analyzed and plotted using the PatchMaster (HEKA Elektronik GmbH, Lambrecht, Germany) and GraphPad Prism 9.0 software (GraphPad Software, San Diego, CA, USA). The current traces were generated using the IGOR Pro software (WaveMetrics, Lake Oswego, OR, USA). Data are presented as mean ± SEM. Tail currents (4 μg WT, 2 μg WT, 4 μg L693P, and 2 μg WT + 2 μg L693P) and Western blot data (WT, L693P, WT/L693P) were compared by one‐way ANOVA with Dunnett's post hoc test versus WT. Kinetic parameters were compared between WT and WT/L693P using unpaired Student's t‐tests. The significance level was set at *p* < 0.05.

## 3 Results

### 3.1 Clinical Characteristics and Genetic Mutation Analysis of the Patient

**FIGURE 1:**
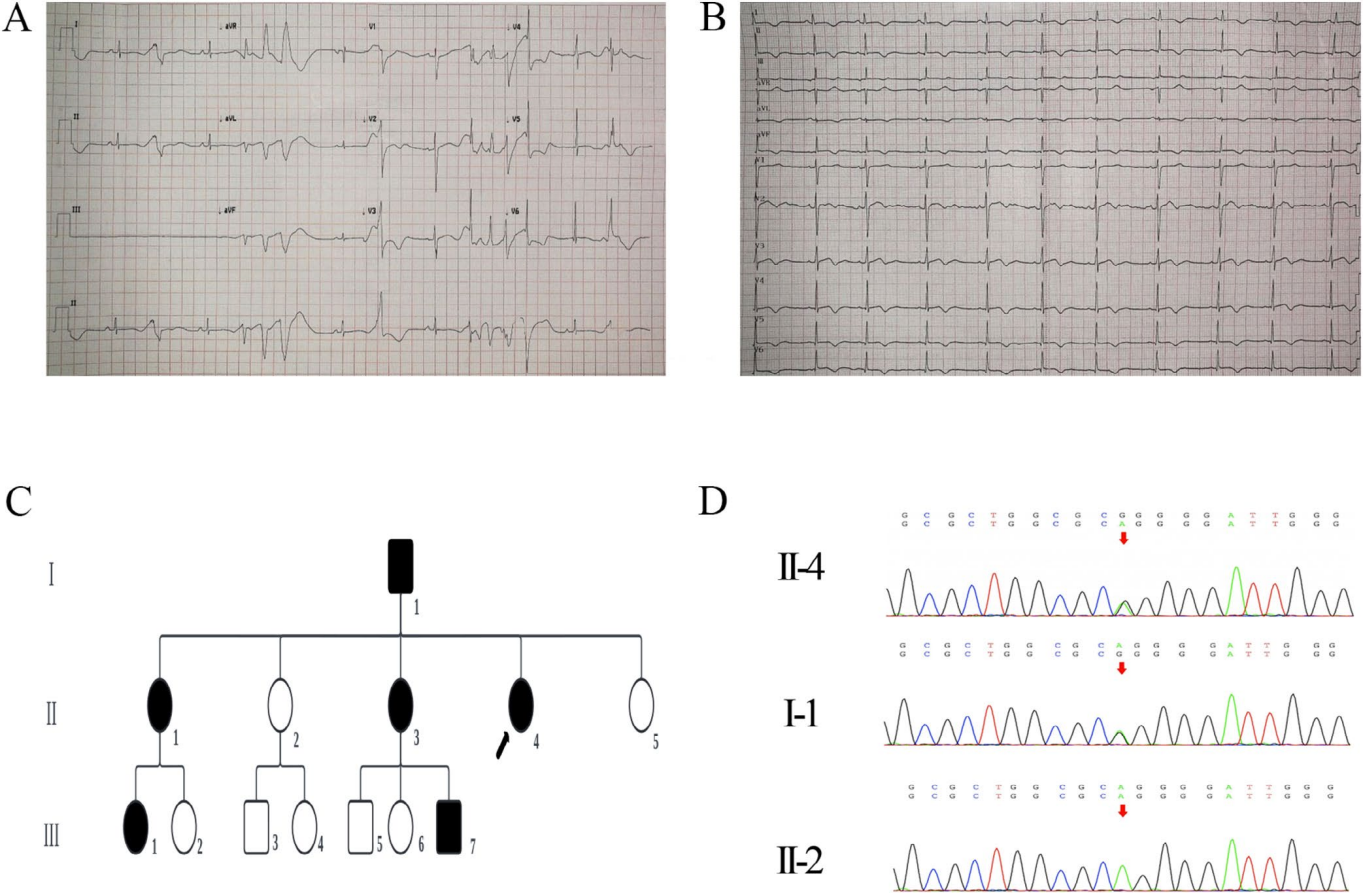
Clinical and genetic characteristics of the proband and family members. (A) Electrocardiogram (ECG) recorded after the proband's most recent syncopal episode at a local hospital, showing frequent multiform premafture ventricular contractions with markedly prolonged QT interval (QTc 600 ms). (B) Follow‐up ECG obtained at our center demonstrating QT prolongation (QTc 530 ms) with bifid T waves. (C) Pedigree of the proband's family. Squares represent males and circles represent females. Filled black symbols indicate carriers of the L693P mutation, open symbols indicate unaffected individuals, and the arrow denotes the proband. (D) Representative Sanger sequencing chromatograms showing the c.2078 T>C (p.Leu693Pro) variant in different family members. The proband (II‐4) and her father (I‐1) are heterozygous carriers of the mutation, indicated by overlapping peaks (red arrows), whereas the proband's sister (II‐2) displays the wild‐type sequence at the same position.

The proband was a 28‐year‐old female (individual II‐4) who presented to the Department of Cardiology at Yan'an Hospital of Kunming Medical University with a history of five recurrent syncopal episodes. Her first syncope occurred at the age of 14, followed by subsequent episodes at 19, 22, and 28 years of age. The syncopal events typically occurred in the early morning or during emotional stress, and occasionally after alcohol intake. Routine biochemical examinations, including serum electrolytes and thyroid function tests, were within normal ranges (Table 1). Echocardiography revealed normal cardiac structure and function. After her most recent syncopal episode, an electrocardiography performed at a local hospital showed frequent multiform premature ventricular contractions and QT prolongation (QTc 600 ms) (Figure 1A). On presentation to our center, repeat electrocardiography demonstrated a markedly prolonged QT interval (QTc 530 ms) accompanied by bifid T waves (Figure 1B). Based on these findings, the proband was diagnosed with congenital long QT syndrome (LQTS) and was started on β‐blocker therapy.

**TABLE 1:** Electrolyte and thyroid function results for the proband during hospitalization.

Parameters	Units	Patient values
Electrolytes		
Potassium (K^+^)	mmol/L	4.14
Sodium (Na^+^)	mmol/L	138.9
Calcium (Ca^2+^)	mmol/L	2.46
Magnesium (Mg^2+^)	mmol/L	0.86
Thyroid function		
TSH	μIU/mL	0.96
Free T4	ng/dL	1.45

We performed clinical evaluation and genetic testing in 13 family members across three generations. Whole exome sequencing of the proband identified a heterozygous *KCNH2* c.2078T>C variant. Subsequent Sanger sequencing confirmed this variant in the proband (II‐4) and five additional family members (I‐1, II‐1, II‐3, III‐1, and III‐7) (Figure 1C,D). This missense variant resulted in the substitution of leucine by proline at codon 693 (p.Leu693Pro). According to the American College of Medical Genetics and Genomics (ACMG) guidelines and the clinical genetic testing report, this variant was classified as a variant of uncertain significance (VUS; ACMG class 3, criteria PM2 + PP3).

**FIGURE 2:**
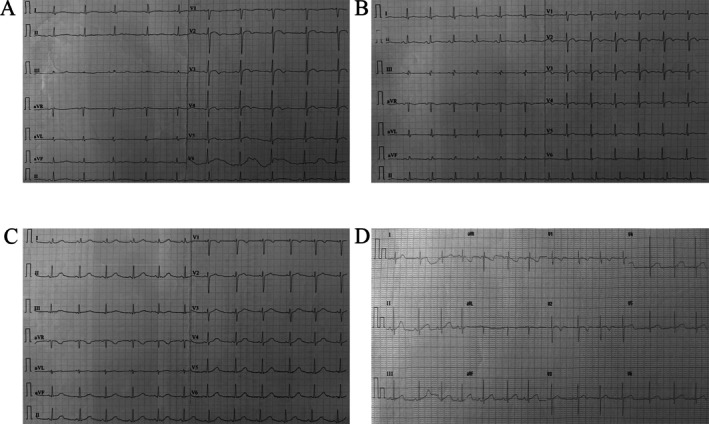
Electrocardiographic abnormalities in family members carrying the KCNH2‐L693P mutation. (A) ECG of the proband's father (I‐1) showing QTc prolongation (470 ms) with bifid T waves. (B) ECG of the proband's aunt (II‐3) demonstrating QTc prolongation (465 ms) with bifid or flattened T waves. (C) ECG of the proband's niece (III‐1) revealing QTc prolongation (480 ms) with bifid T waves. (D) ECG of the proband's nephew (III‐7) showing QTc prolongation (460 ms) with bifid T waves.

Clinical assessments revealed that individual I‐1 (the proband's father) experienced five syncopal episodes since the age of 16, occurring at 16, 20, 22, 26, and 28 years, typically in the early morning or after alcohol consumption. His electrocardiogram showed a prolonged QTc of 470 ms with bifid T waves (Figure 2A), while echocardiography, serum electrolytes, and thyroid function were normal. Individual II‐3 had four syncopal episodes beginning at age 10, with recurrences at 13, 16, and 19 years, which were also predominantly triggered in the early morning. Her electrocardiogram showed QT prolongation (QTc 465 ms) with flattened or bifid T waves (Figure 2B), and her biochemical and echocardiographic findings were unremarkable. Individual II‐1 carried the mutation but remained asymptomatic, and her electrocardiogram was normal. Individuals III‐1 and III‐7 were also asymptomatic but exhibited prolonged QT intervals (QTc 480 ms and 460 ms, respectively) with abnormal T‐wave morphologies (Figure 2C,D). The remaining non‐carrier family members (II‐2, II‐5, III‐2, III‐3, III‐4, III‐5, and III‐6) had no history of syncope, and their electrocardiograms, serum electrolytes, thyroid function, and echocardiographic findings were all normal.

### 3.2 Western Blot and Immunofluorescence Analysis of the *KCNH2‐* L693P Mutation

**FIGURE 3:**
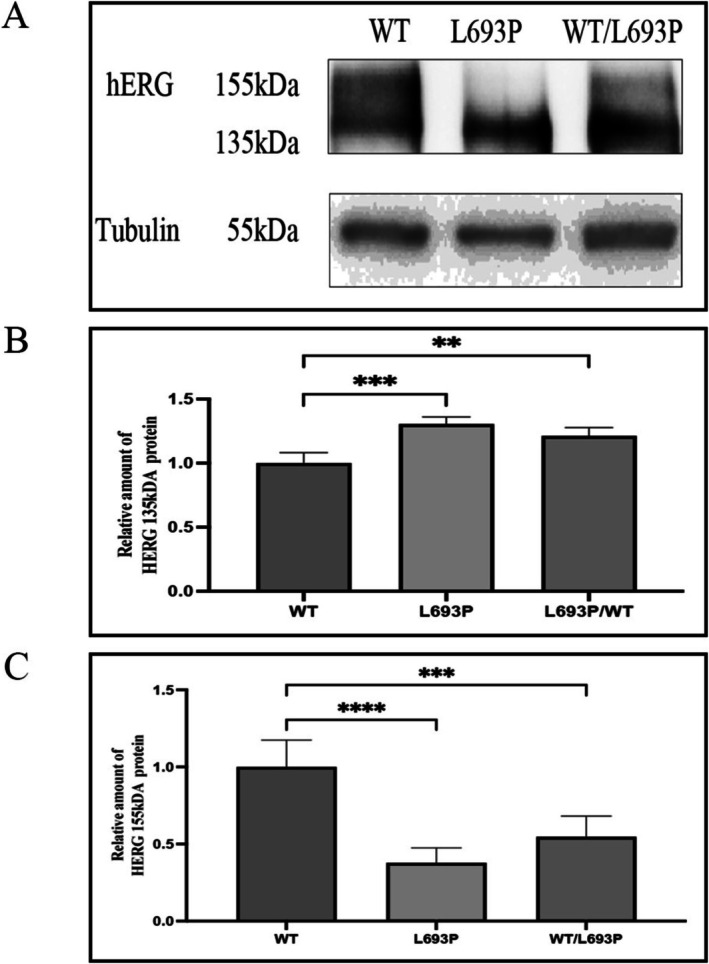
Expression and maturation of hERG protein in WT‐hERG, L693P‐hERG, and WT/L693P‐L693P cells. (A) Representative Western blot showing hERG protein expression and maturation status. The upper band at 155 kDa corresponds to the fully glycosylated mature hERG protein localized to the plasma membrane, whereas the lower band at 135 kDa represents the core‐glycosylated immature hERG protein retained in the endoplasmic reticulum (ER). Mature 155 kDa bands are clearly detected in WT‐hERG and WT/L693P‐hERG groups but are absent in the L693P‐hERG group. Tubulin (55 kDa) served as the loading control. Each lane was loaded with 30 μg of protein. (B) Quantitative analysis of the relative expression levels of the 135 kDa hERG protein in WT, L693P, and WT/L693P cells (*n* = 3). **p < 0.01 vs. WT; ***p < 0.001 vs. WT. (C) Quantitative analysis of the relative expression levels of the 155 kDa hERG protein in WT, L693P, and WT/L693P cells (*n* = 3). ***p < 0.001 vs. WT; ****p < 0.0001 vs. WT.

HEK293 cells were transiently transfected with WT‐hERG, WT/L693P‐hERG, and L693P‐hERG plasmids. After 48 h, total cellular proteins were extracted and analyzed by western blotting. As shown in Figure 3A–C, the wild‐type (WT) hERG protein demonstrated two distinct bands at approximately 135 kDa and 155 kDa, corresponding to the immature and mature forms, respectively. The 135 kDa immature form was localized primarily in the endoplasmic reticulum (ER), whereas the mature 155 kDa form represented the glycosylated form of hERG and was localized in the Golgi apparatus and plasma membrane. Quantitative analysis revealed that the level of the mature 155 kDa band was significantly reduced in the WT/L693P‐hERG group compared with WT (0.55 ± 0.13 vs. 1.00 ± 0.17, *p* < 0.001), and was completely absent in the L693P‐hERG group (*p* < 0.0001). In contrast, the relative amount of the immature 135 kDa band was significantly increased in both the L693P‐hERG (1.31 ± 0.05 vs. 1.00 ± 0.08, *p* < 0.001) and WT/L693P‐hERG groups (1.21 ± 0.06 vs. 1.00 ± 0.08, *p* < 0.01) compared with WT, while no significant difference was observed between L693P‐hERG and WT/L693P‐hERG. These findings indicate that the L693P mutation is associated with impaired hERG protein maturation, characterized by reduced expression of the fully glycosylated 155 kDa form and concomitant accumulation of the immature 135 kDa form, consistent with trafficking defects.

**FIGURE 4:**
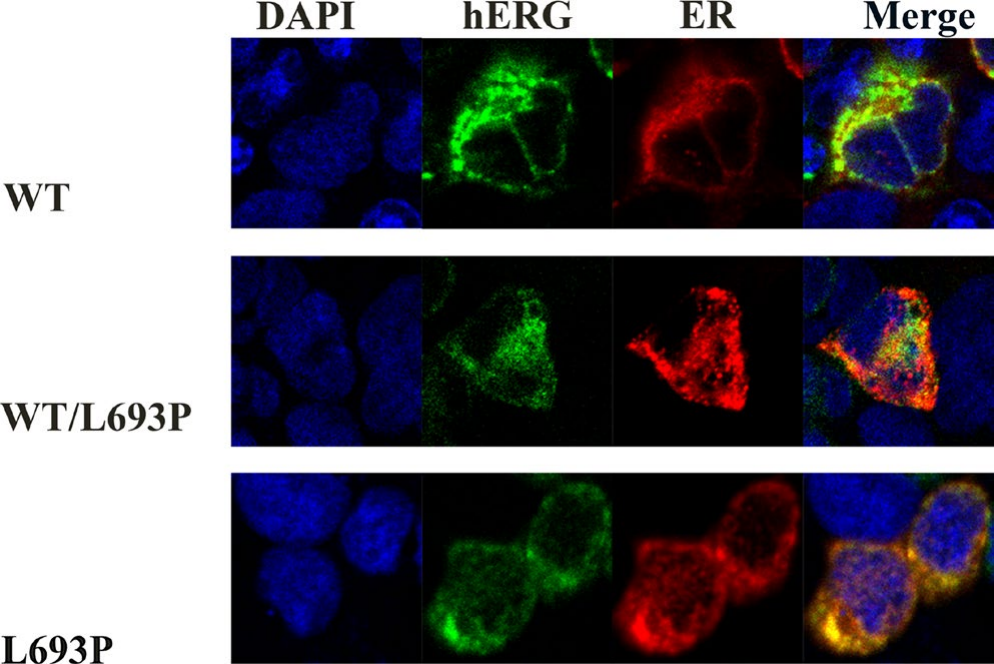
Subcellular localization of hERG protein in WT, L693P, and WT/L693P cells. Confocal microscopy images of HEK293 cells transfected with WT‐KCNH2‐GFP, L693P‐KCNH2‐GFP, or WT/L693P‐KCNH2‐GFP plasmids together with pDsRed2‐ER plasmids. The first column shows nuclear staining with DAPI (blue); the second column shows GFP‐labeled hERG protein (green); the third column shows the ER marker pDsRed2‐ER (red); and the fourth column shows merged images. Row 1: WT group; Row 2: WT/L693P group; Row 3: L693P group. In WT cells, hERG protein is predominantly localized to the plasma membrane. In WT/L693P cells, hERG is mainly retained in the endoplasmic reticulum with partial trafficking to the plasma membrane. In contrast, in L693P cells, hERG protein is almost exclusively retained in the endoplasmic reticulum with no detectable membrane localization.

To further evaluate the trafficking defects, HEK293 cells were co‐transfected with plasmids expressing hERG (GFP) and the ER marker protein pDsRED2‐ER (RFP). Immunofluorescence results (Figure 4) showed minimal overlap of the WT‐hERG (green fluorescence) with the ER marker protein (red fluorescence), thereby indicating its normal trafficking to the plasma membrane. In the WT/L693P‐hERG group, we observed limited membrane localization and partial retention of the hERG within the ER indicated by a significant yellow fluorescence overlap. In the HEK293 cells expressing the homozygous L693P‐hERG mutation, we observed significant ER retention with prominent yellow fluorescence, thereby indicating a severe trafficking defect that prevented its plasma membrane localization.

### 3.3 Functional Analysis of the *KCNH2‐* L693P Channel Mutation

We then transfected HEK293 cells with 4 μg WT‐hERG, 2 μg WT‐hERG, 4 μg L693P‐hERG, or 2 μg WT + 2 μg L693P‐hERG. After 48 h, we performed functional analysis of the ion channels in the cell membrane.

**FIGURE 5:**
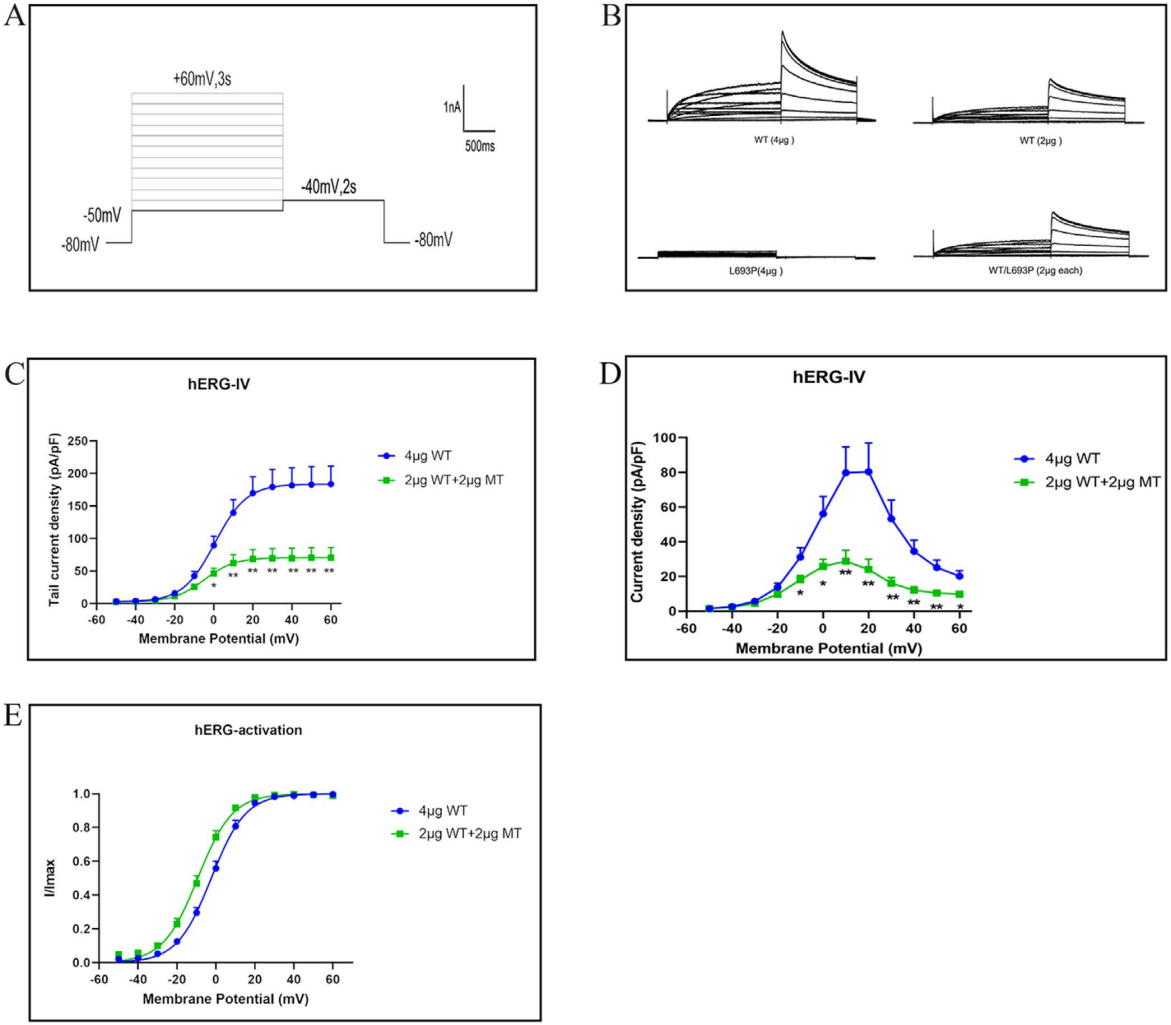
Whole‐cell patch‐clamp analysis of membrane current and channel activation kinetics. (A) Voltage protocol to assess the effects of the L693P mutation on hERG current and activation kinetics. Membrane potential was clamped at −80 mV and subjected to stepwise pulses from −50 mV to +60 mV (increments of 10 mV) for 3 s, followed by a repolarization pulse at −40 mV for 2 s. Sampling frequency was 20.0 kHz, with a filter frequency of 2.9 kHz and a stimulus interval of 15 s. (B) Currents recorded from HEK293 cells transfected with 4 μg WT‐KCNH2, 2 μg WT‐KCNH2, 2 μg WT‐KCNH2 + 2 μg L693P‐KCNH2, and 4 μg L693P‐KCNH2 according to the described voltage protocol. (C‐D) I‐V curves of tail currents (C) and peak currents (D) recorded from the HEK293 cells transfected with 4 μg WT‐KCNH2 (circles) and 2 μg WT‐KCNH2 + 2 μg L693P‐KCNH2 (squares) (*p < 0.05 vs. WT 4 μg, **p < 0.01 vs. WT 4 μg). (E) Steady‐state activation curves obtained by fitting the normalized hERG tail currents with the Boltzmann equation. WT, wild‐type; MT, L693P mutant.

All tested cell membranes were clamped at −80 mV and stimulated with voltage pulses ranging from −50 mV to +60 mV with 10 mV increments for 3 s, followed by a −40 mV pulse for 2 s. The stimulation interval was 15 s (Figure 5A). As shown in Figure 5B, HEK293 cells transfected with WT‐hERG exhibited typical hERG potassium currents, whereas HEK293 cells transfected with the mutant L693P‐hERG did not show any detectable current. When WT‐hERG and L693P‐hERG were co‐transfected into the HEK293 cells, the recorded hERG potassium currents exhibited a current waveform that was comparable to that of WT‐hERG. Table 2 shows the tail current amplitudes of cells transfected with different plasmids. The current amplitude of the HEK293 cells co‐transfected with 2 μg each of WT‐hERG and L693P‐hERG was significantly lower than that of the HEK293 cells transfected with 4 μg of WT‐hERG but was similar to that of the HEK293 cells transfected with 2 μg of WT‐hERG (WT‐hERG vs. WT/L693P‐hERG, 183.67 ± 27.49 pA/pF vs. 70.91 ± 15.26 pA/pF; *p* < 0.05; Figure 5C). This suggested that the L693P‐hERG mutation led to a loss of hERG channel function and exerted a dominant‐negative‐like inhibitory effect.

**TABLE 2:** Activation parameters for the WT‐hERG and L693P‐hERG channel currents.

	WT‐hERG (4 μg) *n* = 8	WT‐hERG (2 μg) *n* = 7	WT/L693P (2 μg) *n* = 8	L693P (4 μg) *n* = 8
Peak tail current (pA/pF)	183.67 ± 27.49	99.54 ± 11.99**	70.91 ± 15.26**	ND
V_1/2_ (mV)	−13.83 ± 1.79	−13.32 ± 1.62	−17.24 ± 1.97	
k (mV)	7.70 ± 0.23	7.31 ± 0.23	7.32 ± 0.32	

Abbreviation: ND, not detectable under experimental conditions.

*p < 0.05 vs. WT 4 μg; **p < 0.01 vs. WT 4 μg.

Next, steady‐state activation was analyzed by plotting voltage (*x*‐axis) versus the ratio of tail current at a given voltage to the maximum tail current (*y*‐axis). The tail currents of WT‐hERG and WT/L693P‐hERG were normalized and fitted with the Boltzmann equation to obtain the steady‐state activation curve of the hERG potassium channel. As shown in Figure 5E, the hERG potassium current activation began at approximately −40 mV and reached near‐complete activation at +20 mV. The half‐activation voltages (V1/2) of WT‐hERG and WT/L693P‐hERG were −13.83 ± 1.79 mV and −17.24 ± 1.97 mV, respectively (*p* > 0.05). The maximum slope factor (*k*) values for WT‐hERG and WT/L693P‐hERG were 7.70 ± 0.23 mV and 7.32 ± 0.32 mV, respectively (*p* > 0.05). These data demonstrated that co‐transfection of WT‐hERG with L693P‐hERG did not alter the activation properties of the WT‐hERG potassium channels.

**FIGURE 6:**
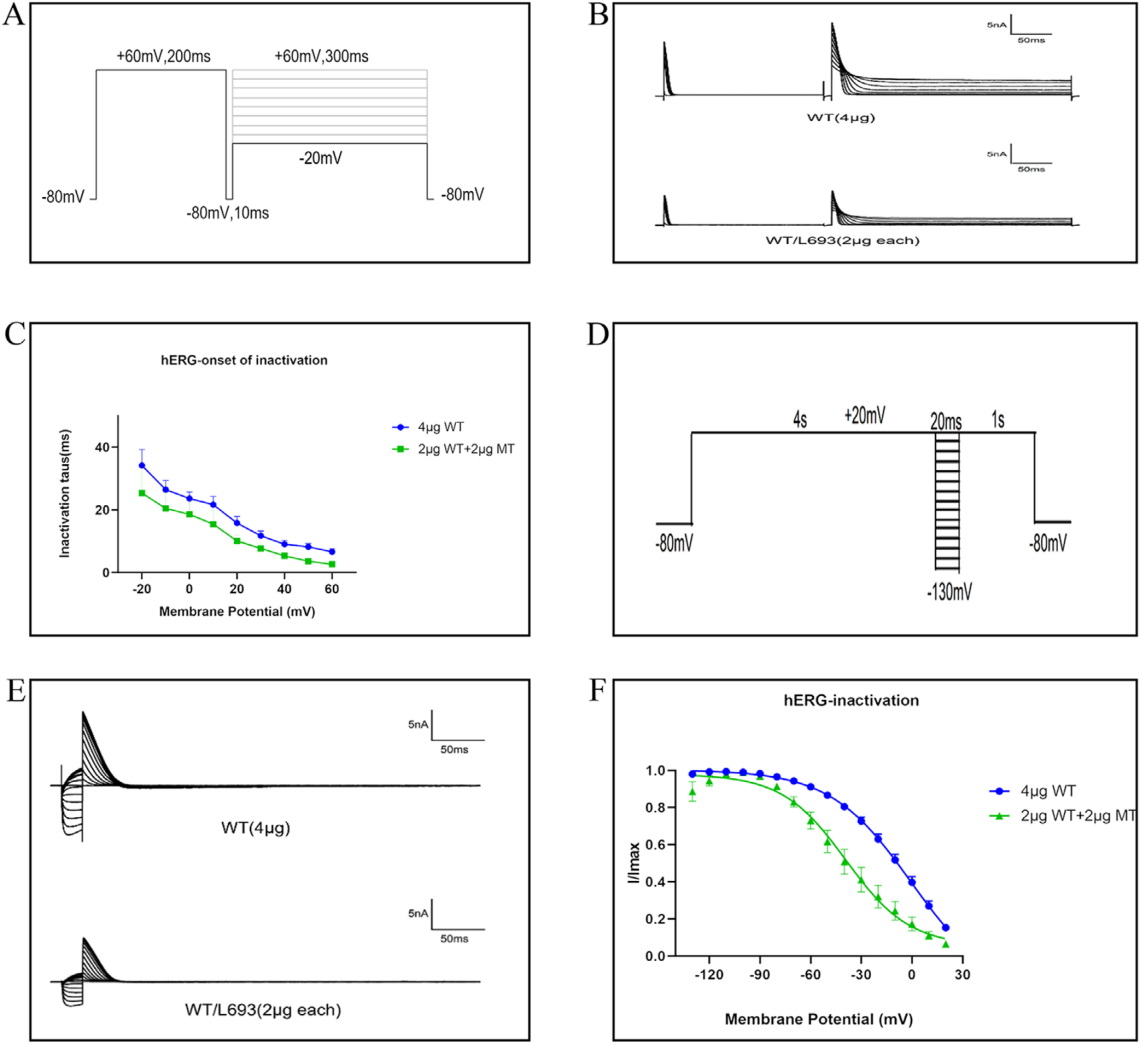
Effects of L693P mutation on channel inactivation and steady‐state inactivation kinetics. (A) Voltage clamp protocol for channel inactivation. Membrane potential was held at −80 mV, depolarized to +60 mV for 200 ms for activation and subsequent inactivation of potassium channels, then hyperpolarized to −80 mV for 10 ms, followed by pulse stimulation from −20 mV to +60 mV (increments of 10 mV) lasting 300 ms. (B) Currents recorded using the inactivation voltage clamp protocol from HEK293 cells transfected with 4 μg WT‐KCNH2 and 2 μg WT‐KCNH2 + 2 μg L693P‐KCNH2. (C) Voltage dependence of channel inactivation time constants from single exponential fitting in cells transfected with 4 μg WT‐KCNH2 (circles) and 2 μg WT‐KCNH2 + 2 μg L693P‐KCNH2 (squares). (D) Voltage clamp protocol for steady‐state inactivation. Membrane potential was clamped at −80 mV, initially depolarized to +20 mV for 4 s for slow activation, followed by inactivation. Cells were then subjected to test pulses from −130 mV to +20 mV (increments of 10 mV) for 20 ms, followed by a third depolarization to +20 mV at 0.1 Hz stimulation frequency. (E) Currents recorded using a steady‐state inactivation protocol. (F) Steady‐state inactivation curves obtained by Boltzmann fitting of normalized currents at different voltages from cells transfected with 4 μg WT‐KCNH2 (circles) and 2 μg WT‐KCNH2 + 2 μg L693P‐KCNH2 (triangles). WT, wild‐type; MT, L693P mutant.

To assess the effects of the L693P‐hERG mutation on potassium channel inactivation, we clamped the cell membrane potential at −80 mV, followed by depolarization to +60 mV for 200 ms, hyperpolarization to −80 mV for 10 ms, and then a series of voltage pulses ranging from −20 mV to +60 mV in 10 mV increments for 300 ms (Figure 6A). The current traces for both WT‐hERG and WT/L693P‐hERG are shown in Figure 6B, WT/L693P‐hERG exhibited smaller current amplitudes than the WT‐hERG. A single exponential function was used to model the inactivation time constants, and the inactivation time constant–voltage relationship was plotted to evaluate the voltage dependence of channel inactivation. The inactivation time constant versus voltage curve (Figure 6C) showed that the inactivation time constant of WT/L693P‐hERG was lower than that of WT‐hERG, but the difference was not statistically significant (*p* > 0.05). This suggested that co‐transfection of WT‐hERG with L693P‐hERG did not affect the instantaneous inactivation process of WT‐hERG.

To evaluate the effect of the L693P‐hERG mutation on steady‐state inactivation, we clamped the cell membrane potential at −80 mV followed by depolarization to +20 mV for 4 s to allow slow activation and subsequent inactivation of the potassium channels. This was followed by a series of voltage pulses from −130 mV to +20 mV in 10 mV increments for 20 ms. The cells were subjected to a third depolarization pulse to +20 mV, which was applied at a frequency of 0.1 Hz (Figure 6D). The steady‐state inactivation current traces of the WT‐hERG and WT/L693P‐hERG channels are shown in Figure 6E. The half‐inactivation voltages (V1/2) of WT‐hERG and WT/L693P‐hERG were 4.24 ± 6.68 mV and −36.39 ± 7.53 mV, respectively (*p* < 0.05), and the slope factors (*k*) were 17.28 ± 6.56 mV and 17.26 ± 1.56 mV, respectively (*p* > 0.05) (Table 3). These results suggested that the L693P‐hERG mutation primarily affected the voltage dependence of the hERG channel inactivation. This caused the L693P‐hERG mutant hERG channels to inactivate at more negative membrane potentials, thereby altering the steady‐state inactivation kinetics. However, this mutation did not significantly affect the rate or steepness of the inactivation process. Therefore, the functional alterations caused by the L693P‐hERG mutation primarily manifested in the voltage dependence of inactivation.

**TABLE 3:** Steady‐state inactivation parameters for the WT‐hERG and L693P‐hERG channel currents.

	WT‐hERG (4 μg) *n* = 8	WT/L693P (2 μg) *n* = 7
V_1/2_ (mV)	4.24 ± 6.68	−36.39 ± 7.53**
k (mV)	17.28 ± 6.56	17.26 ± 1.56

**p < 0.01 vs. WT 4 g.

**FIGURE 7:**
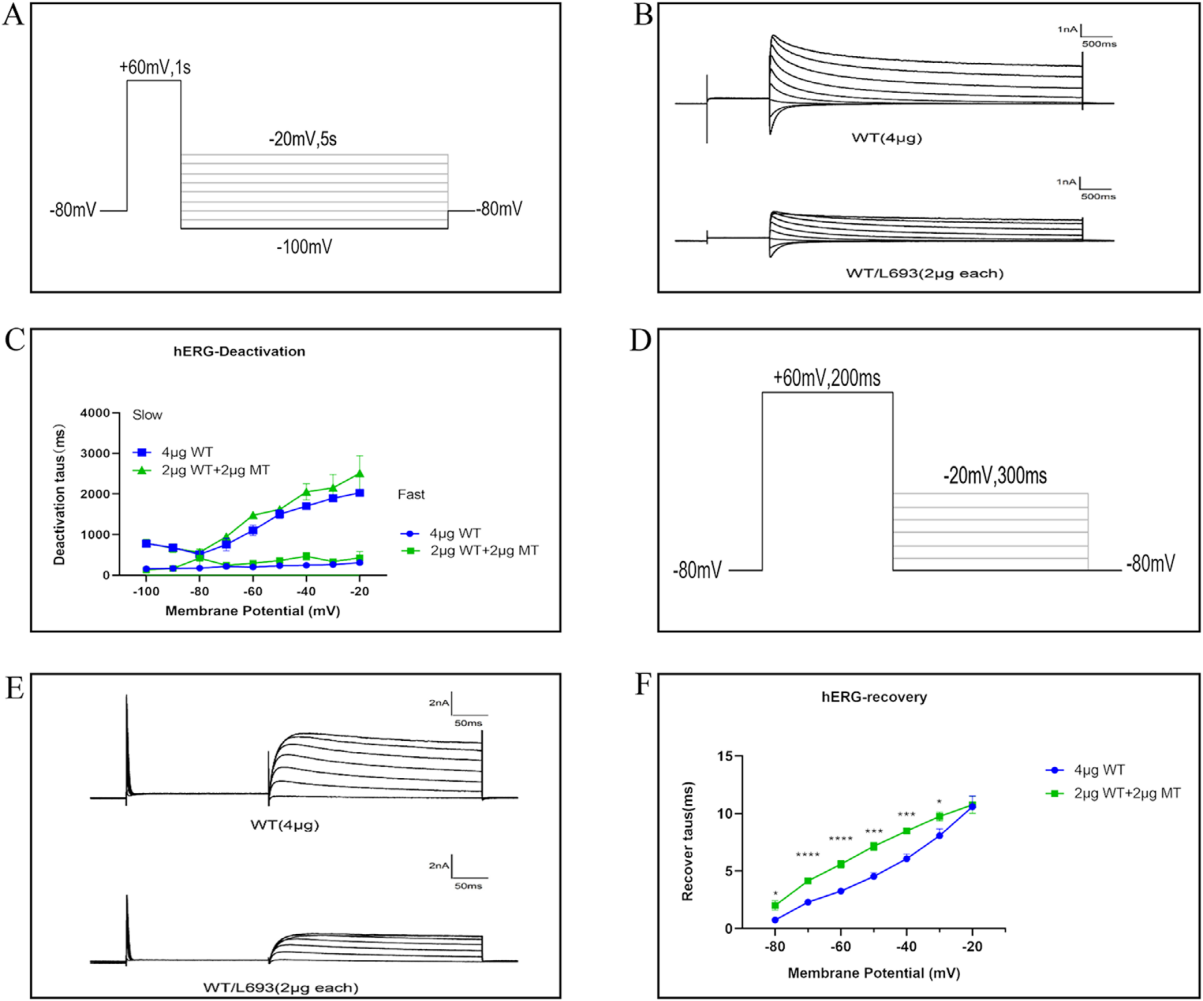
Effects of L693P mutation on the deactivation and recovery kinetics. (A) Voltage clamp protocol for channel deactivation. Membrane potential was held at −80 mV, depolarized to +60 mV for 1 s to fully activate tail currents, followed by pulses from −100 mV to −20 mV (increments of 10 mV) for 5 s, and then repolarized to −80 mV. (B) Currents recorded using the deactivation protocol. (C) Voltage dependence of deactivation time constants (fast and slow components) in cells transfected with 4 μg WT‐KCNH2 (fast‐blue circles, slow‐blue squares) and 2 μg WT‐KCNH2 + 2 μg L693P‐KCNH2 (fast‐green squares, slow‐blue triangles). (D) Voltage‐clamp protocol for channel recovery. Membrane potential was clamped at −80 mV, depolarized to +60 mV for 200 ms to activate and inactivate potassium channels, then subjected to pulses from −80 mV to −20 mV (increments of 10 mV) for 300 ms. (E) Currents recorded under the recovery protocol. (F) Recovery time constants derived from single exponential fitting plotted against voltage for 4 μg WT‐KCNH2 (circles) and 2 μg WT‐KCNH2 + 2 μg L693P‐KCNH2 (squares). *p < 0.05 vs. WT 4 μg; **p < 0.01 vs. WT 4 μg; ***p < 0.001 vs. WT 4 μg; ****p < 0.0001 vs. WT 4 μg.

To assess the effect of the L693P‐hERG mutation on channel deactivation, we clamped the membrane potential at −80 mV, followed by depolarization to +60 mV for 1 s to fully activate the tail current. Then, we applied a series of voltage pulses ranging from −100 mV to −20 mV in 10 mV increments for 5 s followed by repolarization to −80 mV (Figure 7A). The deactivation currents were fitted using a biexponential equation to estimate the time constants for the fast and slow components of current decay. The deactivation time constant–voltage curve was generated by plotting the time constants at each voltage against the corresponding test potentials (Figure 7C). The results showed that hERG potassium channel deactivation was voltage‐dependent, and the co‐transfection of L693P‐hERG with WT‐hERG did not affect the deactivation process of the WT‐hERG potassium channels (*p* > 0.05).

To assess the impact of the L693P‐hERG mutation on recovery from inactivation, we clamped the membrane potential at −80 mV, followed by depolarization to +60 mV for 200 ms to activate and inactivate potassium channels, and a subsequent series of voltage pulses from −80 mV to −20 mV in 10 mV increments for 300 ms (Figure 7D). The recovery currents were fitted using a single exponential equation, and the recovery time constants were plotted as a function of voltage (Figure 7F) and are summarized in Table 4. Compared with WT‐hERG, WT/L693P‐hERG channels exhibited significantly prolonged recovery time constants at −80 to −30 mV, indicating that the L693P mutation slows recovery from inactivation and delays the transition back to the activated state.

**TABLE 4:** Recovery time constants (*τ*) of WT‐hERG and WT/L693P‐hERG channels at different test voltages.

Voltage (mV)	WT‐hERG (4 μg)	WT/L693P‐hERG (2 μg)
−80	0.7450 ± 0.1941	2.009 ± 0.4104*
−70	2.290 ± 0.2007	4.139 ± 0.2539****
−60	4.526 ± 0.3318	5.594 ± 0.3317*
−50	4.526 ± 0.3318	7.154 ± 0.3506****
−40	6.064 ± 0.4231	8.481 ± 0.2869***
−30	8.074 ± 0.5875	9.743 ± 0.3896*
−20	10.60 ± 0.9301	10.76 ± 0.7411

*p < 0.05 vs. WT 4 μg; ***p < 0.001 vs. WT 4 μg; ****p < 0.0001 vs. WT 4 μg.

## 4 Discussion

Congenital long QT syndrome (LQTS) is a cardiac disease caused by genetic mutations affecting the heart's electrical activity. It is primarily characterized by QT interval prolongation on an electrocardiogram (ECG), which increases the risk of potentially fatal arrhythmias (Ono et al. 2020). Type 2 congenital long QT syndrome (LQT2) is specifically caused by mutations in the *KCNH2* gene. Previous studies have demonstrated four primary pathogenic mechanisms for LQT2 caused by *KCNH2* mutations. Class I mutations lead to inhibition of hERG protein synthesis or translation by generating incomplete proteins or triggering nonsense‐mediated RNA decay (NMD) (Gong et al. 2007; Sanguinetti et al. 1996; Stump et al. 2013). Class II mutations, the most common cause of LQT2, lead to misfolded proteins that are retained significantly in the endoplasmic reticulum (ER) by the cellular quality control systems because of improper folding, thereby disrupting the trafficking of the channel proteins to the cell membrane (Ficker et al. 2000; Han et al. 2014; Liu et al. 2013). Class III mutations alter the gating properties of the ion channels (Chen et al. 1999; Nakajima et al. 1998; Zhou et al. 1998). Class IV mutations affect the ionic permeation/selectivity of the channels (Lees‐Miller et al. 2000). These mechanisms prolong the repolarization phase of the action potential and increase the risk of arrhythmia.

Because of significant advances in high‐throughput sequencing technologies, more than 2000 *KCNH2* mutation sites have been documented in public databases (www.uniprot.org). Some *KCNH2* mutations have been verified by western blot and/or whole‐cell patch clamp experiments, thereby confirming their pathogenic mechanisms and providing evidence for clinical management. However, most of the *KCNH2* variants have not been verified at a molecular level. Furthermore, sequencing companies often lack comprehensive clinical information to interpret the mutations and rely on internal algorithms to categorize variants as pathogenic, benign, or variants of uncertain significance (VUS) (Ackerman et al. 2016). Therefore, approximately 10% of LQTS‐related variants are incorrectly classified (Kapa et al. 2009; Refsgaard et al. 2012). As previously reported, clinicians accepting reports from genetic testing companies without evidence‐based judgment could lead to unnecessary invasive treatments or delayed interventions (Ackerman et al. 2016). Therefore, accurately distinguishing between pathogenic and benign mutations is critically important.

A patient experiencing recurrent syncope was admitted to our hospital. The patient exhibited a significantly prolonged QT interval on ECG, but did not have a history of medications, thyroid hormone abnormalities, or electrolyte imbalances. Based on the Schwartz scoring criteria, the patient's score exceeded 4 points, leading to the diagnosis of congenital long QT syndrome (Schwartz et al. 1993; Zeppenfeld et al. 2022). Subsequently, based on the whole exome sequencing results, we identified a missense mutation (p.Leu693Pro) in the *KCNH2* gene. According to the ACMG guidelines, this variant was classified as a VUS. Due to the discrepancy between genotype and phenotype, we encountered difficulties in subsequent treatment and management decisions. Based on the principle of “phenotype is king, genotype is queen,” we ultimately considered the VUS to be pathogenic (Ackerman 2015). To validate its pathogenicity, we performed in vitro experiments with the HEK‐293 cell line.

The validation experiments demonstrated that the *KCNH2‐*L693P mutation caused significant functional impairments. Western blot analyses showed impaired glycosylation of the mutant hERG protein harboring the *KCNH2‐*L693P mutation. Quantitative analysis revealed a significant reduction or complete loss of the fully glycosylated 155 kDa mature form in mutant groups, whereas the relative level of the immature 135 kDa form was significantly increased. This pattern is consistent with a Class II trafficking defect, where misfolded hERG proteins are retained within the ER and fail to undergo complex glycosylation (Anderson et al. 2006; Lamothe et al. 2016). Importantly, the accumulation of the 135 kDa band reflects impaired maturation rather than enhanced synthesis, indicating that the cellular quality control system prevents aberrant hERG proteins from reaching the plasma membrane (Foo et al. 2019). Although the precise physiological consequence of 135 kDa accumulation remains unclear, it may exacerbate ER stress, activate unfolded protein response pathways, or reduce the functional channel density at the membrane, ultimately contributing to arrhythmogenesis (Foo et al. 2019). Immunofluorescence experiments further confirmed this result and showed that the mutant hERG protein was trapped in the ER because of trafficking defects (Ma et al. 2019).

Electrophysiological experiments demonstrated that the *KCNH2*‐L693P mutation significantly reduced the current amplitudes of the heterozygous hERG channels and exhibited dominant‐negative‐like suppression on the wild‐type (WT)‐hERG channel function (Liu et al. 2013; Mihic et al. 2011; Owusu‐Mensah et al. 2024). Moreover, the L693P‐hERG mutation significantly slowed down the recovery rate of the heterozygous hERG channels from the inactivated to activated state (Amin et al. 2008). Collectively, these findings demonstrated that the *KCNH2‐*L693P mutation prolonged the repolarization phase of the action potentials in cardiomyocytes by impairing the trafficking and kinetics of the heterozygous hERG channels. This aligned with the characteristics of the Class II pathogenic mutations in LQT2. Integrating the above experimental results and the clinical features of the proband, our data supported that this variant was pathogenic. Therefore, the *KCNH2‐*L693P mutation warrants re‐evaluation and reclassification as a clinically significant pathogenic mutation. Furthermore, our findings provide robust evidence guiding therapeutic decision‐making for patients harboring this mutation.

Despite clarifying the significant pathogenic effects of the *KCNH2‐*L693P mutation on the heterozygous hERG channel function, our study has a few limitations. The main limitation is that the functional analyses in this study were exclusively performed in the HEK293 cells. Therefore, future experimental validation in cardiomyocytes is required to confirm our findings because they would closely mimic physiological conditions.

## 5 Conclusions

Our study demonstrates that the *KCNH2‐*L693P mutation is a pathogenic variant. Based on our results, this mutant should be reclassified from a variant of uncertain significance to a pathogenic variant in genetic testing reports. This mutation leads to reduced expression of the mutant hERG protein on the cell membrane because of defective protein glycosylation and impaired trafficking. Functionally, the L693P‐hERG mutation significantly suppressed ion channel tail currents in the heterozygous state, leaving only ~39% of the WT current. This degree of suppression, which is more severe than simple haploinsufficiency but not a complete dominant‐negative block, is consistent with a dominant‐negative‐like interference with WT channel function. In addition, the mutation caused abnormalities in steady‐state inactivation and recovery kinetics from inactivation. Taken together, these findings demonstrate that the *KCNH2‐*L693P mutation played an essential role in the pathogenesis of congenital long QT syndrome type 2, thereby emphasizing the need for targeted clinical management and intervention for individuals carrying this mutation.

## Author Contributions

All authors made a significant contribution to the work reported, whether that is in the conception, study design, execution, acquisition of data, analysis and interpretation, or in all these areas; took part in drafting, revising or critically reviewing the article; gave final approval of the version to be published; have agreed on the journal to which the article has been submitted; and agree to be accountable for all aspects of the work.

## Data Availability

The data that support the findings of this study are available from the corresponding author upon reasonable request.
